# Genome-Wide Analysis of β-Galactosidases in *Xanthomonas campestris* pv. *campestris* 8004

**DOI:** 10.3389/fmicb.2018.00957

**Published:** 2018-05-11

**Authors:** Huiqi Wang, Chenyi Shi, Qingbiao Xie, Yaxin Wang, Shiyao Liu, Chunxia Li, Chaozu He, Jun Tao

**Affiliations:** ^1^Hainan Key Laboratory for Sustainable Utilization of Tropical Bioresources, Haikou, China; ^2^Institute of Tropical Agriculture and Forestry, Hainan University, Haikou, China

**Keywords:** β-galactosidase, glycosyl hydrolase (GH) family 2 and 35, LacZ, reporter gene, *Xanthomonas campestris* pv. *campestris*

## Abstract

Bacterial β-galactosidase is involved in lactose metabolism and acts as a prevalent reporter enzyme used in studying the activities of prokaryotic and eukaryotic promoters. *Xanthomonas campestris* pv. *campestris* (*Xcc*) is the pathogen of black rot disease in crucifers. β-Galactosidase activity can be detected in *Xcc* culture, which makes *Escherichia coli* LacZ unable to be used as a reporter enzyme in *Xcc*. To systemically understand the β-galactosidase in *Xcc* and construct a β-galactosidase -deficient strain for promoter activity analysis using LacZ as a reporter, we here analyzed the putative β-galactosidases in *Xcc* 8004. As glycosyl hydrolase (GH) family 2 (GH2) and 35 (GH35) family enzymes were reported to have beta-galactosidase activities, we studied all of them encoded by *Xcc* 8004. When expressed in *E. coli*, only two of the enzymes, XC1214 and XC2985, were found to have β-galactosidase activity. When deleted from the *Xcc* 8004 genome, only the *XC1214* mutant had no β-galactosidase activity, and other GH2 and GH35 gene deletions resulted in no significant reduction in β-galactosidase activity. Therefore, XC1214 is the main β-galactosidase in *Xcc* 8004. Notably, we have constructed a β-galactosidase-free strain that can be employed in gene traps using LacZ as a reporter in *Xcc*. The results reported herein should facilitate the development of high-capacity screening assays that utilize the LacZ reporter system in *Xcc*.

## Introduction

β-Galactosidase is widely distributed across species and is important as a key provider in the production of energy and a source of carbon through the breakdown of lactose to galactose and glucose (Rodríguez et al., 2006; Juers et al., 2012). The enzyme is frequently used in genetics, molecular biology, and other life sciences as it can efficiently hydrolyze X-gal (5-bromo-4-chloro-3-indoyl-β-D-galactopyranoside), a soluble, colorless compound, into galactose and 5-bromo-4-chloro-3-hydroxyindole, which is then oxidized into 5,5'-dibromo-4,4'-dichloro-indigo, an insoluble blue product that makes bacteria encoding β-galactosidase become blue on growth plates containing X-gal (Gary and Kindell, 2005; Smale, 2010; Juers et al., 2012). β-Galactosidase activity can also be easily detected in solution by using ortho-nitrophenyl-β-galactoside (ONPG) as a substrate. As the product ortho-nitrophenol is yellow (λ_max_ = 420 nm), enzyme activity can be measured by the rate of appearance of a yellow color using a spectrophotometer (A_420_) (Smale, 2010).

*Xanthomonas campestris* pv. *campestris* (*Xcc*) is the pathogen of black rot disease, which is considered the most destructive disease of crucifers, including all cultivated varieties of brassicas worldwide (Vicente and Holub, 2013). *Xcc* can become blue when growing on a medium containing X-gal and thus expresses β-galactosidase (Fu and Tseng, 1990). There are only three annotated galactosidase genes (designated *galA, galB*, and *galC*) in the *Xcc* genome of 8004 and ATCC 33913 strains (da Silva et al., 2002; Qian et al., 2005). GalA and GalB are similar to glycosyl hydrolase (GH) family 2 enzymes and have no detectable activities in *Xcc* Xc17 (Yang et al., 2003). GalC is a GH35 enzyme homologous to the *X. axonopodis* pv. *manihotis* Bga and has β-galactosidase activity in *Xcc* Xc17 (Yang et al., 2003). Another galactosidase, GalD, has been reported to be a member of the GH35 family and exhibits a low level of β-galactosidase activity in *Xcc* Xc17 (Yang et al., 2007). Mutations of *galA, galB*, and *galC* did not reduce the ability to grow on lactose or the β-galactosidase activity in *Xcc* Xc17L, a strain with elevated β-galactosidase activity, indicating that these three genes do not encode the main β-galactosidase in Xc17L (Yang et al., 2003). An Xc17L *galD* mutant had the same β-galactosidase activity as Xc17, suggesting that GalD may be a β-galactosidase (Yang et al., 2007). However, the identity of the major β-galactosidase in *Xcc* remains unclear.

In this study, we analyzed the GH2 and GH35 family proteins in *Xcc* 8004 and compared their β-galactosidase activities in *Xcc* cells or expression in *E. coli*. We also constructed a β-galactosidase-free strain that can be employed in gene traps using LacZ as a reporter to detect the regulation of gene expression in *Xcc*.

## Materials and methods

### Bacterial strains and growth conditions

The bacterial strains and plasmids used in this work are listed in Table S1. *Xcc* strains were grown at 28°C in the rich medium NYG or synthetic medium M4 (Daniels et al., 1984; Tao et al., 2014). *E. coli* strains were grown in LB medium at 37°C (Tao et al., 2014). Antibiotics were used at the following final concentrations, as required: 100 μg/ml ampicillin (Amp), 50 μg/ml kanamycin (Kan), 100 μg/ml rifampicin (Rif) and 100 μg/ml spectinomycin (Sp).

### DNA manipulation and vector construction

PCR cloning, restriction enzyme digestion, agarose gel electrophoresis, and SDS-PAGE have been discussed elsewhere (Tao et al., 2014). PCR primers and the application are listed in Table S2. All constructs were verified by sequencing.

### Generation of *Xcc* deletion mutants

Primer design, suicide vector construction, and mutant screening were conducted as in a previous report (Tao et al., 2014). Briefly, approximately 500 bp sequences flanking both sides of the region to be deleted were amplified by PCR and cloned into pK18mobSacB. *Xcc*-competent cells were transformed with suicide plasmids by electroporation and plated on NYG with kanamycin. After two rounds of recombination, the open reading frame was deleted from the genomic DNA. Mutants were confirmed by PCR and DNA sequencing.

### β-galactosidase activity assay

β-Galactosidase activity was determined as previously described (Zhang and Bremer, 1995; Cromie et al., 2006; Smale, 2010). Briefly, cells were cultured in LB liquid media (37°C, 200 rpm). When the OD_600_ reached 0.8, the cells in 1 ml were harvested by centrifugation at 4°C. The supernatant was discarded, and the cells were resuspended in 1 ml of Z buffer (60 mM Na_2_HPO_4_, 40 mM NaH_2_PO_4_, 10 mM KCl, 1 mM MgSO_4_, and 50 Mm β-mercaptoethanol). The cells were lysed by sonication. Until the mixture became clear, to ensure complete cell lysis, the reaction was started by adding 0.2 ml of ONPG (4 mg/ml). The reaction was stopped by adding 0.5 ml of Na_2_CO_3_ (1 M) stock solution when the sample in the tube developed a pale yellow color. The OD_420_ of the reactions was measured. The assays were performed in triplicate, and one miller unit (U) was expressed as 1,000 × OD_420_/(OD_600_ of assaying culture × volume assayed × time).

In plate assays, bacteria were spotted on LB (for *E. coli*) or NYG (for *Xcc*) plates supplemented with 20 mg/l X-gal. For assays for β-galactosidase mutants, each spot was inoculated with 2 μl of a 10-fold dilution series (i.e., 10^−2^-, 10^−3^-, 10^−4^-, and 10^−5^-fold from left to right) from cells in logarithmic growth phase (OD_600_ = 0.8).

### Extracellular enzyme activity test

*Xcc* strains were cultivated in NYG medium supplemented with xylan, cellulose, starch, skimmed milk, and β-mannose (Tang et al., 1991; Qian et al., 2008). If a mutant has the ability to secrete the enzyme, a hydrolysis zone will be produced around the colony (Qian et al., 2008). By comparing the size of D/d [diameter of the hydrolysate (D)/colony diameter (d)] to identify strains with enzyme production capacity, the larger the value of D/d, the stronger was the ability of the strain to produce enzymes.

A single colony was picked and cultured in 5 ml of NYG liquid medium; 1 ml of culture (OD_600_ = 0.8) was centrifuged to collect the cells. Pellets were washed twice with ddH_2_O and resuspended in 100 μl of NYG. Next, 1 μl of the suspension was spotted onto an NYG solid plate with 0.2% RBB-xylan (Sigma), 0.5% carboxymethyl cellulose (CMC, Sigma), 0.1% soluble starch, 2% skim milk powder or 0.5% locust bean gum for the analysis of xylanase, cellulose, amylase, protease, and mannanase activities, respectively.

### Ability of sugar utilization

A single colony was picked and cultured in 5 ml of NYG liquid medium, and 1 ml of the culture was centrifuged to collect the cells. Pellets were washed twice with 1 ml ddH_2_O and resuspended in 100 μl of M4 media. Next, 1 μl of the suspension was spotted onto an M4 solid plate containing 1% glucose, fructose, glycerol, lactose, galactose, sucrose, or maltose. The growth status of the mutants and the ability to utilize sugar were photographed.

### Bacterial disease assays

The virulence of *Xcc* for cabbage (*Brassica oleracea*) was tested as described previously (Qian et al., 2005). Briefly, bacterial cells (OD_600_ = 0.5) were introduced into the leaves by leaf clipping. Twelve leaves were used, and the lesion length was measured 14 days postinoculation.

### Bioinformatics analysis

Amino acid sequence alignment was conducted with the ClustalX program (Larkin et al., 2007). The putative β-galactosidase sequences in *Xcc* 8004 were downloaded from GenBank (accession no. CP000050). A bootstrapped neighbor joining tree was constructed with MEGA 6 software based on the sequence alignment by the ClustalX program (Larkin et al., 2007; Tamura et al., 2013). The conserved domains were predicted by the CDD search (Marchler-Bauer et al., 2017).

### Statistical methods

Data were analyzed by ANOVA using the PROC GLM (*P* ≤ 0.05) procedure in SAS software.

## Results

### *In silico* analysis of the GH2 and GH35 family proteins in *Xcc*

**Figure 1:**
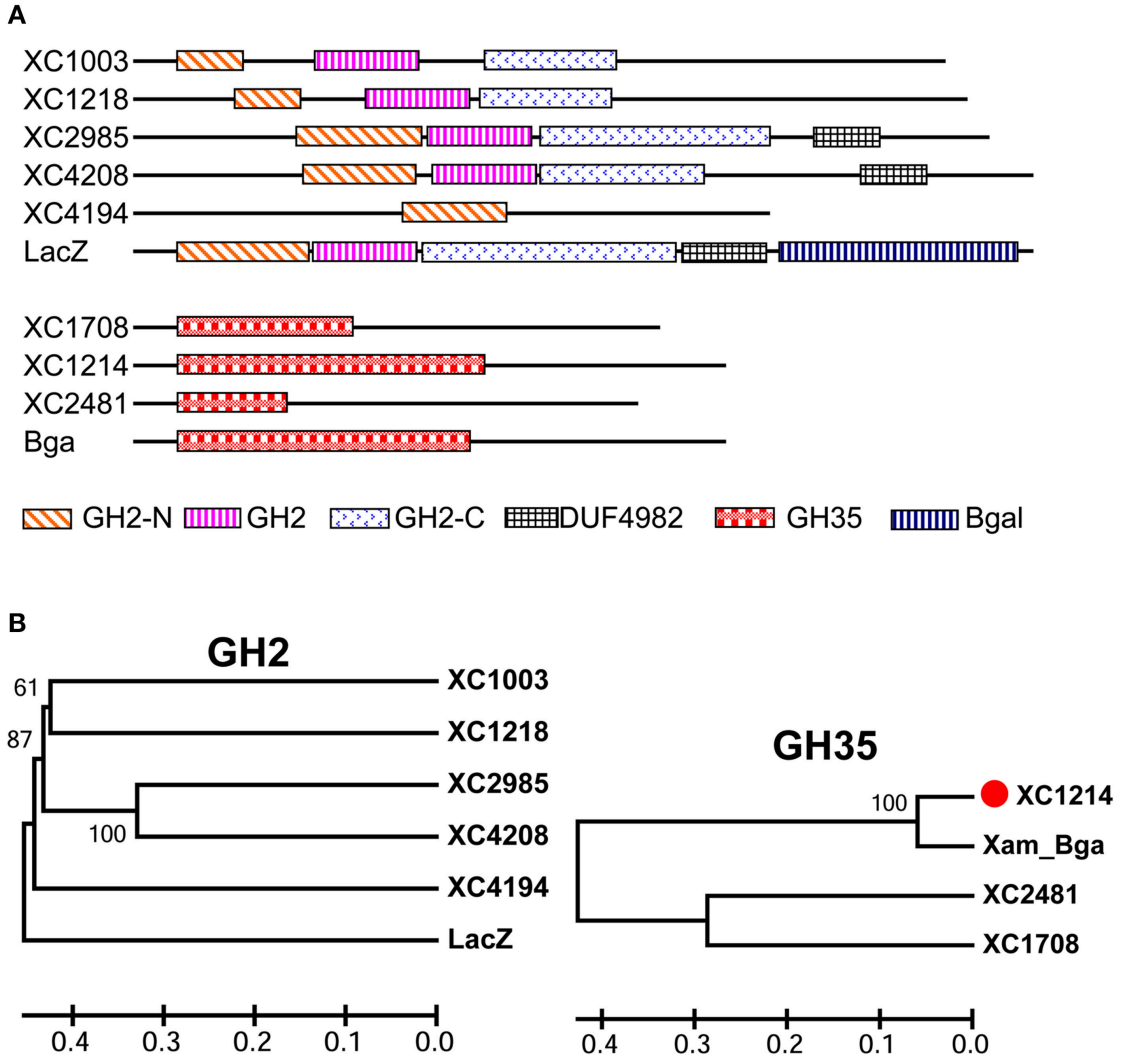
The putative β-galactosidases in *Xcc* 8004. **(A)** The domains in the eight putative β-galactosidases of *Xcc* 8004, the LacZ of *E. coli* and Bga of *X. axonopodis* pv. *manihotis* predicted by CDD-BLAST. **(B)** The evolutionary relationship of the two protein (GH2 and GH35) families of *Xcc* putative β-galactosidases computed with the UPGMA rule in MEGA6 software and full-length proteins. The horizontal axis shows the Nei distance.

In the *Xcc* 8004 genome (Qian et al., 2005), there are five putative genes encoding GH2 proteins and three putative genes for GH35 proteins, according to the CDD-BLAST (Figure 1; Marchler-Bauer et al., 2017). All these proteins have predicated GH2 or GH35 domains in the N-terminus, and most of them have no other known domains (Figure 1A). GH2 proteins have low similarity, but GH35 proteins have high similarity with each other in *Xcc* (Figures 1B and Figure S1). The GH2 and GH35 domains in these proteins are not conserved (Figure S1). Moreover, none of the GH2 proteins in *Xcc* is highly similar (>75%, amino acid) to the best known β-galactosidase, LacZ, in *E. coli*. The GH35 proteins in *Xcc* are homologous to the Bga of *X. axonopodis* pv. *manihotis* (Figures 1B and Figure S1), which is a known β-galactosidase(Yang et al., 2003). These data suggest that GH2 proteins have diverse functions but that GH35 proteins might have β-galactosidase activities in *Xcc*.

### Expression and β-galactosidase activity analysis of the GH2 and GH35 family proteins in *E. coli*

**Figure 2:**
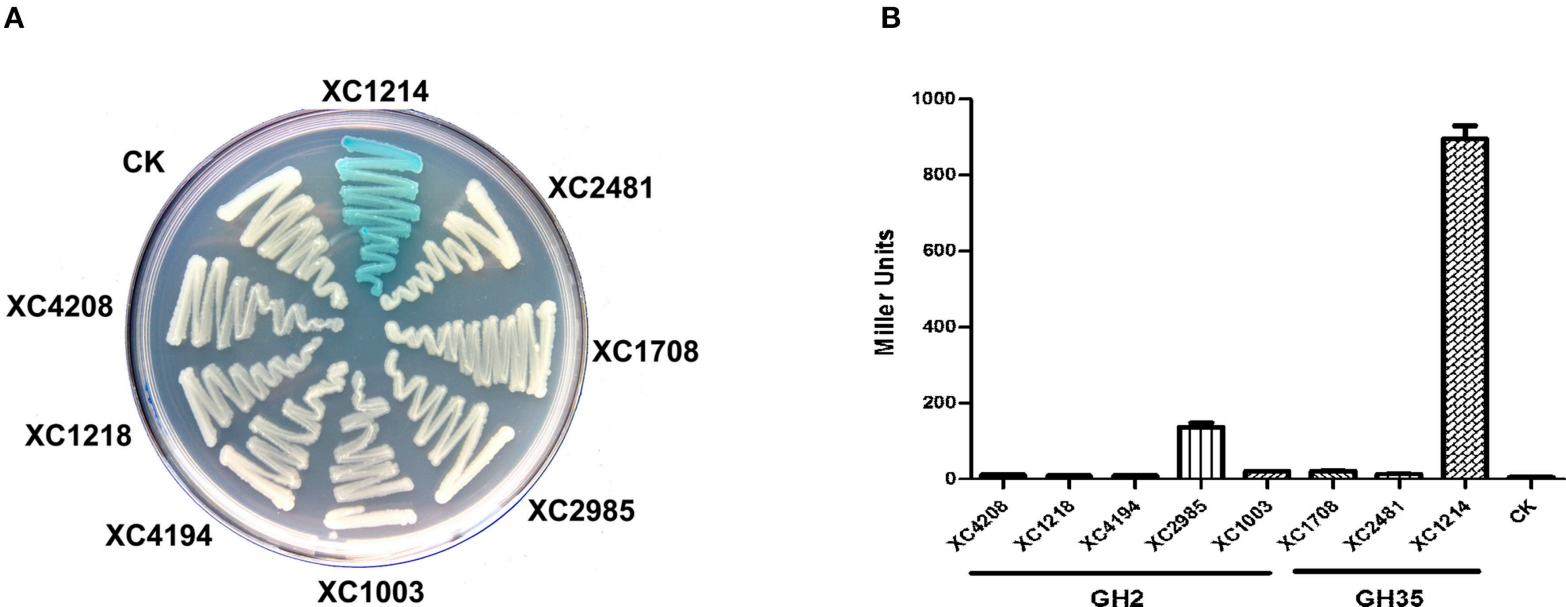
β-galactosidase activities of *Xcc* GH2 and GH35 proteins expressed in *E. coli*. **(A)** Morphology of GH2- and GH35-expressing *E. coli* cultured on X-gal containing media. **(B)** The β-galactosidase activities of GH2- and GH35-expressing *E. coli*. CK: *E. coli* containing the empty vector. Triplicate experiments were performed, and the means ± standard deviations (*SD*s) were calculated.

To analyze the enzyme activities of GH2 and GH35 proteins, we cloned the individual corresponding genes into the expression vector and expressed them in *E. coli* DH5α, which has no β-galactosidase activity. When these strains grew on LB media supplemented with X-gal, only the *XC1214-*expressing strain became blue (Figure 2A), indicating that the other seven proteins might not be β-galactosidases. The β-galactosidase activities of the lysates of the eight gene-expressing cells were also analyzed. The results showed that *XC1214* and *XC2985* had detectable β-galactosidase activities but that the others did not. The activity of *XC2985* was ~137 U, but that of *XC1214* was ~896 U (Figure 2B). All of the genes were sufficiently expressed in *E. coli* (Figure S2), indicating that the activity difference of these strains is not due to the fact that these GH2 and GH35 genes cannot be expressed in *E. coli*. Taken together, our data imply that *XC1214* and *XC2985*, not the other six GH2 and GH35 proteins, might be the β-galactosidase in *Xcc*.

### Mutagenesis analysis of the GH2 and GH35 genes in *Xcc*

**Figure 3:**
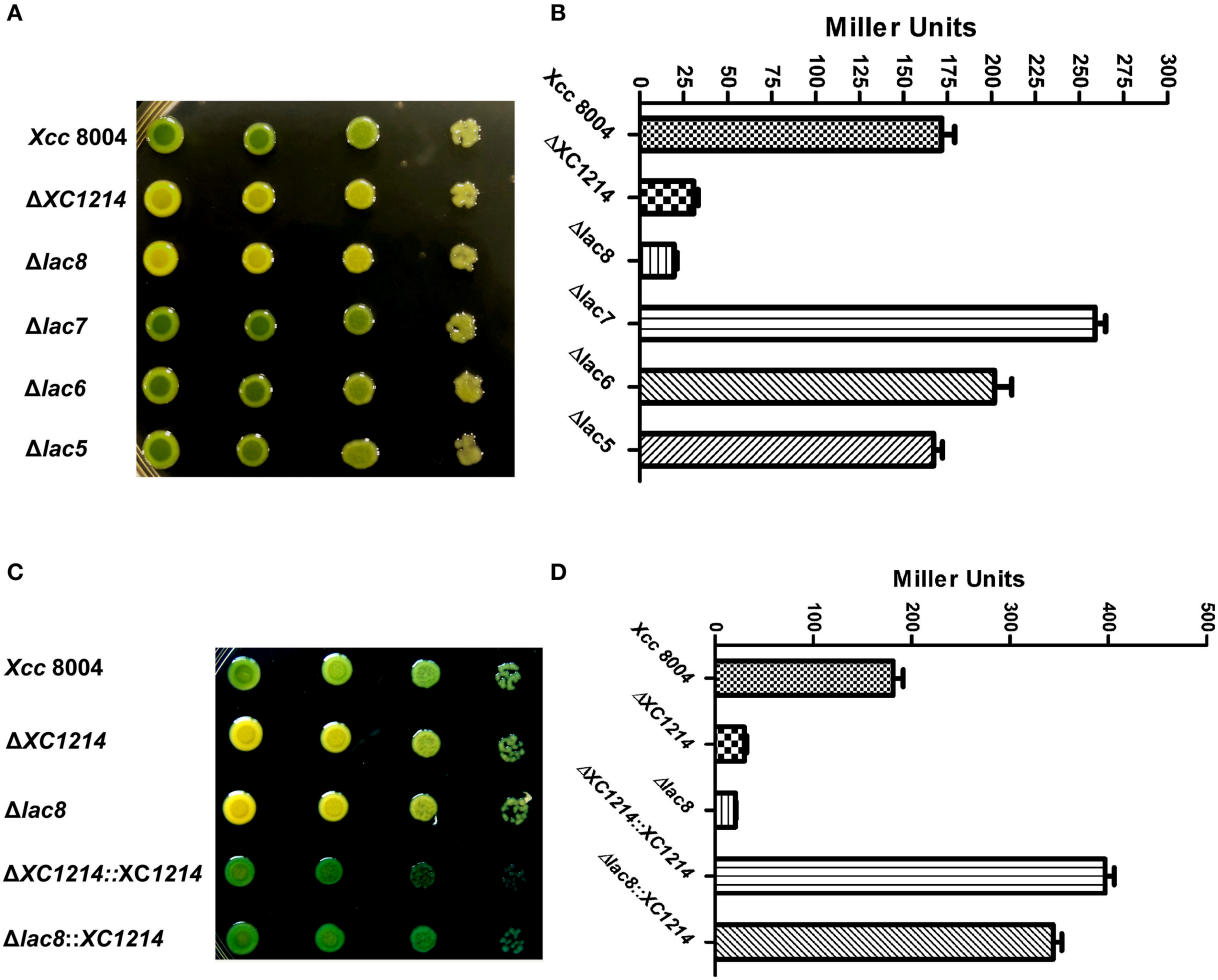
The enzyme activities of GH2 and GH35 proteins in *Xcc* 8004. Morphology of the GH2 and GH35 deletion mutants **(A)** on X-gal containing plates and the β-galactosidase activities of these strains **(B)**. The morphology **(C)** and β-galactosidase activity **(D)** of the *XC1214* mutant and its complementary strain. Each spot was inoculated with 2 μl of a 10-fold dilution series (i.e., 10^−2^-, 10^−3^-, 10^−4^-, and 10^−5^-fold from left to right) from cells in the logarithmic growth phase (OD_600_ = 0.8). Triplicate exper**i**ments were performed, and the means ± *SD*s were calculated.

To systemically analyze the functions of the GH2 and GH35 proteins in *Xcc*, we deleted all eight genes from the *Xcc* 8004 genome. Δ*XC1214* and the mutant (Δ*lac8*) that had neither GH2 nor GH35 genes were not able to turn blue when grown on NYG medium containing X-gal (Figure 3A and Figure S3); the mutant (Δ*lac7*) that was deficient in all these genes except *XC1214* showed a stronger blue color than that of wild-type *Xcc* 8004. The β-galactosidase activity of Δ*lac7* was also higher than that of *Xcc* 8004 (Figure 3B). These data indicate that some of the other GH2 and GH35 genes may negatively regulate *XC1214* expression or XC1214 activity. However, we did not detect a difference of *XC1214* expression in *Xcc* 8004 and Δ*lac7* (data not shown), suggesting that the other GH2 and GH35 proteins may regulate XC1214 activity.

**Figure 4:**
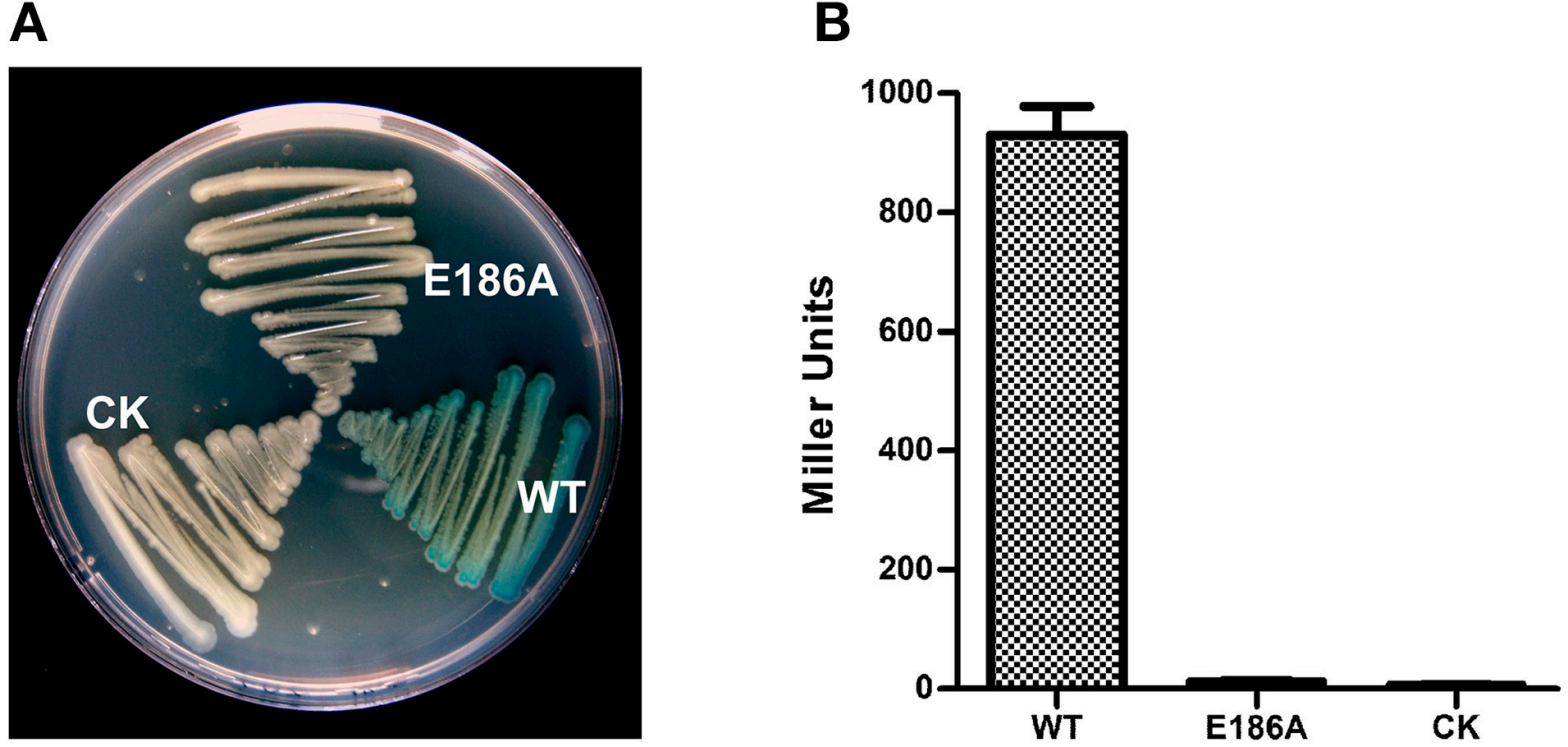
The active site for enzyme activity of the XC1214 protein. Mutation of the active site (E186) of XC1214 resulted in loss of β-galactosidase activity both on plates **(A)** and in liquid media **(B)**. Triplicate experiments were performed, and the means ± *SD*s were calculated.

Because XC2985 expressed in *E. coli* had β-galactosidase activity (Figure 2B), it may also have had activity in *Xcc*. However, the mutant (Δ*lac5*) in which *XC1003/XC2985/XC4208/XC2481/XC1708* was deleted had the same phenotypes as *Xcc* 8004 (Figure 3B). Thus, XC2985 was not the β-galactosidase or was not expressed in *Xcc* under our experimental conditions. We also transformed the individual GH2 and GH35 genes controlled by a strong promoter into Δ*lac8* and detected the β-galactosidase activities of these transformed strains. Only *XC1214* introduction resulted in restored Δ*lac8* β-galactosidase activity (Figures 3C,D). Furthermore, we substituted the alanine for the conserved active amino acid (Glu^186^) of XC1214. This mutation resulted in loss of β-galactosidase activity of XC1214 (Figures 4A,B). Taken together, these data confirmed that XC1214 was the major β-galactosidase in *Xcc* 8004.

### Phenotypic analysis of the GH2 and GH35 mutants

**Figure 5:**
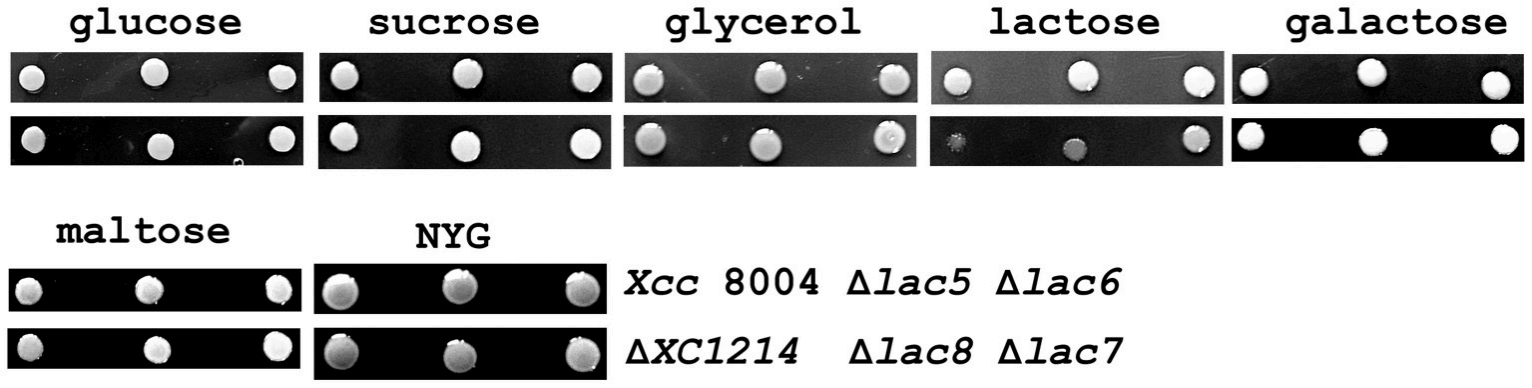
The sugar usage of the GH2 and GH35 mutants in *Xcc* 8004. The growth of the indicated strains (right end panel) on solid plates when the individual sugars were used as the sole carbon source. Δ*lac5*: Δ*XC1003*/Δ*XC2985*/Δ*XC4208*/Δ*XC2481*/Δ*XC1708*; Δ*lac6*: Δ*lac5*/Δ*XC4194*; Δ*lac7*: Δ*lac6*/Δ*XC1218*; Δ*lac8*: Δ*lac7*/Δ*XC1214*.

As discussed above, except for XC1214, the functions of other GH2 and GH35 proteins are unclear. Because β-galactosidase is the essential component for lactose utilization, we wanted to know whether these GH2 and GH35 proteins are also involved in sugar utilization. Therefore, the ability of the bacteria to utilize sugar was studied. We determined whether a given bacterium had the ability to disintegrate a certain saccharide by using glucose, lactose, maltose, β-galactose, sucrose, or glycerol as the sole carbon source. It was found that the mutant strains grew as well as *Xcc* 8004 on the glucose-, sucrose-, galactose-, maltose-, and glycerol-containing plates (Figure 5). When lactose was the sole carbon source, Δ*XC1214* and Δ*lac8* were found to grow poorly compared with *Xcc* 8004 and the other mutants (Figure 5). Therefore, XC1214 was the major enzyme involved in lactose metabolism.

**Figure 6:**
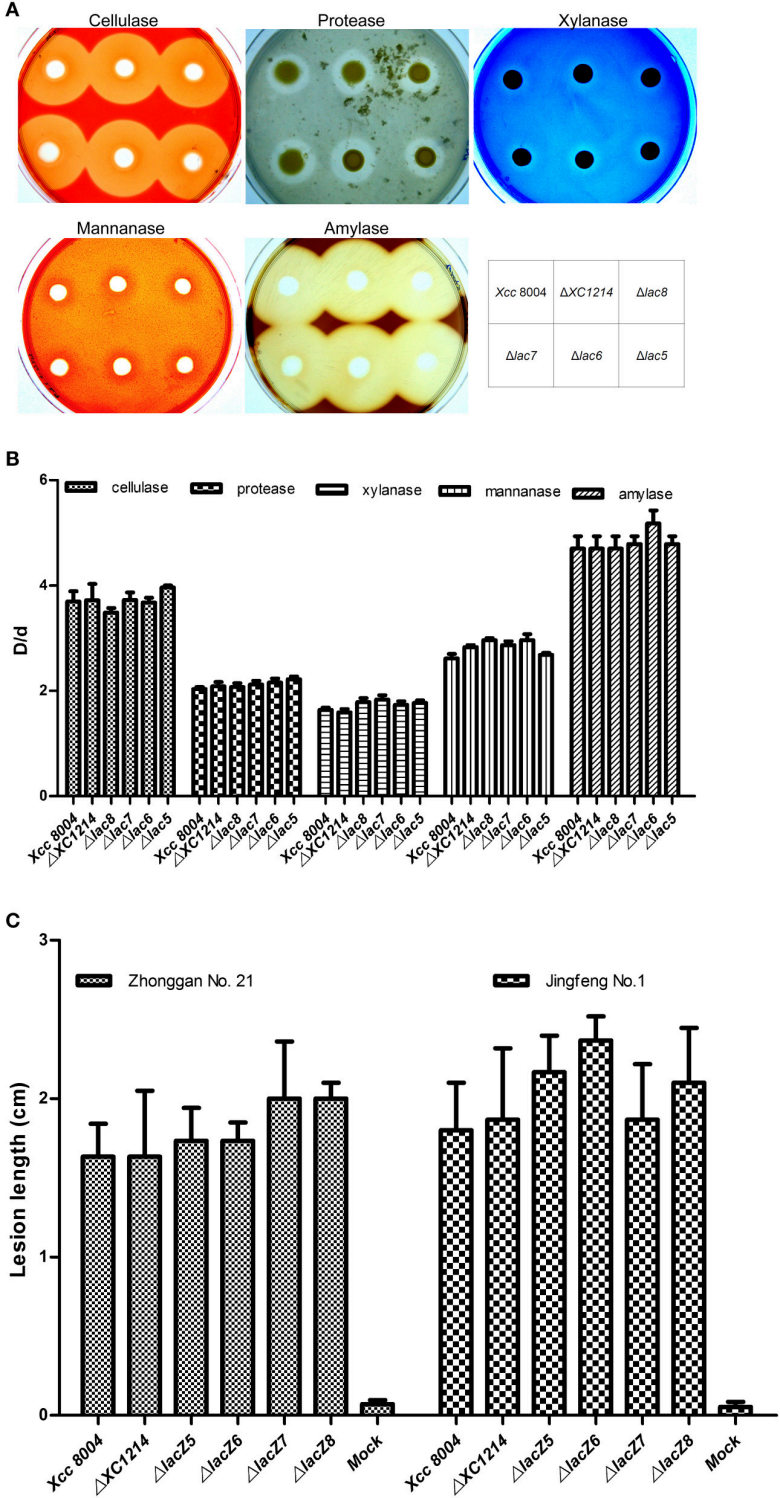
Virulence-related analysis of the GH2 and GH35 mutants. **(A,B)** The extracellular enzyme activities of the GH2 and GH35 mutants. **(C)** The virulence of the GH2 and GH35 mutants on cabbage 9 days after inoculation. Jingfeng No. 1 and Zhonggan No. 21 are two species of *Brassica oleracea*. Virulence assays were performed on 12 leaves, and the means ± *SDs* were calculated.

Many enzymes are secreted into the extracellular compartment to hydrolyze their respective substrates. Thus, we compared the activities of extracellular enzymes such as pectinase, cellulase, protease, amylase, and xylanase in *Xcc* 8004 and in the GH2 and GH35 mutants. The results showed no obvious difference between *Xcc* 8004 and the GH2 and GH35 mutants (Figures 6A,B), indicating that GH2 and GH35 proteins should not have or regulate these extracellular enzyme activities.

To determine whether GH2 and GH35 proteins are involved in pathogenesis, *Xcc* 8004 and the mutants were inoculated onto different varieties of cabbage. The disease symptoms on the leaves were observed 9 days after inoculation. The results showed that there were no significant differences between *Xcc* 8004 and the mutants (Figure 6C), indicating that GH2 and GH35 proteins were not the main virulence factors of *Xcc* 8004.

## Discussion

The *E. coli lacZ* gene encoding β-galactosidase (β-gal) can be used as a standard reporter to monitor promoters or enhancers in transient or stable transfection experiments (Evans et al., 2013; Aviv and Gal-Mor, 2018). *Xcc* 8004 has the property of degrading lactose and forms blue spots on X-GAL plates (Figures 3, 6). Therefore, LacZ cannot be used as a tool for promoter activity analysis in *Xcc* 8004. Therefore, the current reporter system widely used in *Xcc* is based on β-glucuronidase, but this system is time consuming and costly and cannot be directly detected on plates. As the LacZ reporter system is an easy, low-cost, visible and stable detectable method (Smale, 2010; Juers et al., 2012; Aviv and Gal-Mor, 2018), we wanted to construct a β-galactosidase-free strain that could be employed in gene traps using LacZ as a reporter for high-throughput detection of the regulation of gene expression in *Xcc*. To this end, we analyzed the activity of the putative β-galactosidase in *E. coli* and *Xcc*. We found the major β-galactosidase in *Xcc* and constructed a β-galactosidase-deficient strain (Δ*lac8*) that had no difference in virulence, growth, or extracellular enzyme activity compared with the wild-type strain. Therefore, LacZ can be used as a reporter gene for high-throughput screening of promoter activity in the Δ*lac8* strain.

Although previous studies reported several putative β-galactosidase in *Xcc* Xc17 (Fu and Tseng, 1990; Yang et al., 2003, 2007), the genomic analysis was not performed. In this study, we systemically analyzed the putative β-galactosidases (GH2 and GH35 family proteins) in *Xcc* 8004. By analyzing the carbohydrate utilization capacity and β-galactosidase activities of the eight genes, Only XC1214 was found to have detectable enzyme activity in *Xcc*.8004. XC2481 (GalD) has been reported as the β-galactosidase that contributes to the elevated β-galactosidase activity in XC17L (Yang et al., 2007). However, our results showed that XC2481 had no β-galactosidase activity in *E. coli* and *Xcc* 8004. In addition, deletion of *galC* (*XC1214*) did not change the β-galactosidase activity in XC17L (Yang et al., 2003), which is opposite to our results in *Xcc* 8004. The difference may be due to the different *Xcc* strains, but the mechanism should be mined in detail.

What functions of the other seven GH2 and GH35 proteins were not found in this study. One possibility is that these proteins also have β-galactosidase activity in growth environments differing from our test conditions. The other possibility is that these proteins have a novel activity to cope with environmental change during the *Xcc*-environment interaction. In addition to β-galactosidase, GH2 were described to function as β-mannosidase, β-glucuronidase, endo-β-mannosidase, β-glucosidase, or exo-β-glucosaminidase (Schröder et al., 2015). For example, the GH2 protein, NixH, was found to have, β-mannosidase activity (Dupoiron et al., 2015). As low similarity between *Xcc* GH2 proteins and *E. coli* LacZ, XC1003, XC1218, XC4194, and XC4208 in *Xcc* 8004 might act as the enzymes as aforementioned. The GH35 comprises enzymes with the activities of β-galactosidase, galacto-β-D-galactanase, and exo-β-glucosaminidases (Kotake et al., 2005; Gamauf et al., 2007). Thus, XC1708 and XC2481 that had no β-galactosidase activity (Figures 2, 3) might have one of the other activities. To uncover the functions of these GH2 and GH35 proteins in *Xcc* 8004, further chemical and genetic analyses should be addressed in the future work.

## Author contributions

HW, CS, and QX did most of the preparation and characterizations; YW and SL contributed to bacterial growth and enzyme activity assays; CL helped with the experimental data; CH and JT designed the experimental scheme and wrote the manuscript. All authors reviewed the manuscript.

### Conflict of interest statement

The authors declare that the research was conducted in the absence of any commercial or financial relationships that could be construed as a potential conflict of interest.
