# Supplementary material for: Genome-Wide Analysis of β-Galactosidases in Xanthomonas campestris pv. campestris 8004
Source: Front Microbiol. 2018 May 11;9:957. doi: 10.3389/fmicb.2018.00957 (PMC5958218; doi:10.3389/fmicb.2018.00957)
Supplement: Supplementary file 1 [file Table_1.pdf]

Supplementary Tables

Table S1. Bacterial strains and plasmids used in this study

Name	Relevant genotype or description	Reference
Strains		
Xcc		
Xcc 8004	A wild-type *Xcc* strain, a laboratory strain with spontaneous rifampicin-resistance	[1]
Δ*XC1214*	Full length deletion of *XC1214*	This study
Δ*lac5*	Deletion mutant of *XC1003*, *XC2985*, *XC4208*, *XC2481*, *XC1708*	This study
Δ*lac6*	Deletion of *XC4194* in Δ*lac5*	This study
Δ*lac7*	Deletion of *XC1218* in Δ*lac6*	This study
Δ*lac8*	Deletion of *XC1214* in Δ*lac7*	This study
E. coli		
DH5α	F^- Φ80 *lac*ZΔM15 Δ(*lac*ZYA-*arg*F) U169 *rec*A1 *end*A1 *hsd*R17(r_k^-, m_k^+) *pho*A *sup*E44 *thi*-1 *gyr*A96 *rel*A1 λ^-	[2]
Plasmids		
pQE80L	Expression vector, Amp^R	Qiagen
pK18*mobsacB*	Suicide plasmid in *Xanthomonas*, Kan^R	[3]
pHM1	broad-host range vector	[4]
pK18-*XC1003*	pK18*mobsacB* based plasmid for *XC1003* deletion	This study
pK18-*XC2985*	pK18*mobsacB* based plasmid for *XC2985* deletion	This study
pK18-*XC4208*	pK18*mobsacB* based plasmid for *XC4208*deletion	This study
pK18-*XC2481*	pK18*mobsacB* based plasmid for *XC2481* deletion	This study
pK18-*XC1708*	pK18*mobsacB* based plasmid for *XC1708*deletion	This study
pK18-*XC4194*	pK18*mobsacB* based plasmid for *XC4194* deletion	This study

pK18-*XC1218*	pK18*mobsacB* based plasmid for *XC1218* deletion	This study
pK18-*XC1214*	pK18*mobsacB* based plasmid for *XC1214* deletion	This study
pQE-*XC1003*	pQE80L based plasmid for *XC1003* expression in *E. coli*	This study
pQE-*XC2985*	pQE80L based plasmid for *XC2985* expression in *E. coli*	This study
pQE-*XC4208*	pQE80L based plasmid for *XC4208* expression in *E. coli*	This study
pQE-*XC2481*	pQE80L based plasmid for *XC2481* expression in *E. coli*	This study
pQE-*XC1708*	pQE80L based plasmid for *XC1708* expression in *E. coli*	This study
pQE-*XC4194*	pQE80L based plasmid for *XC4194* expression in *E. coli*	This study
pQE-*XC1218*	pQE80L based plasmid for *XC1218* expression in *E. coli*	This study
pQE-*XC1214*	pQE80L based plasmid for *XC1214* expression in *E. coli*	This study
pQE-*XC1003*	pQE80L based plasmid for *XC1003* expression in *E. coli*	This study
pQE-*XC2985*	pQE80L based plasmid for *XC2985* expression in *E. coli*	This study
pQE-*XC4208*	pQE80L based plasmid for *XC4208* expression in *E. coli*	This study
pQE-*XC2481*	pQE80L based plasmid for *XC2481* expression in *E. coli*	This study
pQE-*XC1708*	pQE80L based plasmid for *XC1708* expression in *E. coli*	This study
pQE-*XC4194*	pQE80L based plasmid for *XC4194* expression in *E. coli*	This study
pQE-*XC1218*	pQE80L based plasmid for *XC1218* expression in *E. coli*	This study
pQE-*XC1214*	pQE80L based plasmid for *XC1214* expression in *E. coli*	This study
pQE-*XC1214E186A*	*E186A* mutant of pQE-*XC1214*	This study
pHM-*XC1003*	pHM1 based plasmid for *XC1003* expression in *Xcc*	This study
pHM-*XC2985*	pHM1 based plasmid for *XC2985* expression in *Xcc*	This study
pHM-*XC4208*	pHM1 based plasmid for *XC4208* expression in *Xcc*	This study
pHM-*XC2481*	pHM1 based plasmid for *XC2481* expression in *Xcc*	This study
pHM-*XC1708*	pHM1 based plasmid for *XC1708* expression in *Xcc*	This study

pHM-*XC4194*	pHM1 based plasmid for *XC4194* expression in *Xcc*	This study
pHM-*XC1218*	pHM1 based plasmid for *XC1218*expression in *Xcc*	This study
pHM-*XC1214*	pHM1 based plasmid for *XC1214* expression in *Xcc*	This study

Amp^R and Kan^R indicate resistance to ampicillin and Kanamycin, respectively

Table S2. Oligonucleotide primers used in this study

Name	Sequence(5′-3′)	Application
For deletion		
XC1003ddFF	GGTCAAGCTTGTGCGCATTGCAGGTCTAGGT	*XC1003* deletion
XC1003ddFR	AGCAGGTACCTTCGAGCTGCCCAGCCATCTG	
XC1003ddRF	AGCAGGTACCGAGCCGCACCGTGGAGATTCG	
XC1003ddRR	GGTCGAATTCCGAATCGGTGATGGTGACGTC	
XC2985ddFF	GGTCAAGCTTGCAACGCCAAGCTGTTCAATG	*XC2985* deletion
XC2985ddFR	AGCAGGTACCCAGCGTGTTTTGCAGCTGCTG	
XC2985ddRF	AGCAGGTACCGCAGCGCTGCATCTGCAGGCT	
XC2985ddRR	GGTCGAATTCAGGACAAGCGGTCATCTGCTC	
XC4208ddFF	GGTCAAGCTTGGTGGGCTTCAACGACGACAC	*XC4208*deletion
XC4208ddFR	AGCAGGTACCGGTAGTGCAGAGGCAGATCAG	
XC4208ddRF	AGCAGGTACCCATGTGCAATCCGACACGCTG	
XC4208ddRR	GGTCGAATTCGGCCAACGATGAACATGCCAC	
XC2481ddFF	GGTCAAGCTTCATGTGGCCTACGGCGAAAC	*XC2481* deletion
XC2481ddFR	AGCAGGTACCCAGGGAAGACAACAGCAACG	
XC2481ddRF	AGCAGGTACCTCCAGTGCACCGCAGCTGCTC	
XC2481ddRR	GGTCGAATTCATCGACCACCTGCAGACGCAC	
XC1218ddFF	GGTCAAGCTTCAGGGTCTGCGCCACTGTGTC	*XC1218* deletion
XC1218ddFR	AGCATCTAGAGGGTGCAGGAACACGACGGTG	
XC1218ddRF	AGCATCTAGACGAGAGCAAATGACGTTGCAG	
XC1218ddRR	GGTCGAATTCCCTCGCTCCACCATTCGTAGG	
XC1214ddFF	GGTCAAGCTTGCAACTACGTACTCGGCAGC	*XC1214* deletion
XC1214ddFR	AGCATCTAGACAAGCGTGGTACGCAACATG	
XC1214ddRF	AGCATCTAGACAGCAGCAGGTGTGGATCAC	
XC1214ddRR	GGTCGAATTCCCTGAGCGAGATCGCACCAG	
XC1708ddFF	GGTCAAGCTTGTCGGCATCTATGGTGCCTC	*XC1708* deletion
XC1708ddFR	AGCATCTAGACAGCATGCAGAGCAGCACTT	
XC1708ddRF	AGCATCTAGACCAGCTCAACGGCGACCAGA	
XC1708ddRR	GGTCGAATTCCCAGCACGTCCAACAGGCTG	
XC4194ddFF	GGTCAAGCTTCCACCGTGCTGATCACAACG	*XC4194* deletion
XC4194ddFR	AGCATCTAGATGCCACCCGTGTCCGTGCAC	
XC4194ddRF	AGCATCTAGAGTGCACCTTGTACGACCGCA	
XC4194ddRR	GGTCGAATTCCGTCGCTGGTTTCGGCGATG	
For clone into pQE80L		

XC1003QEF	GATCGCATCACCATCACCATCACGGATCCGCTGGGCAGCTCGAACCGCA	XC1003 gene for expression
XC1003QER	CAGGAGTCCAAGCTCAGCTAATTAAGCTTACACGGCATAGCAACTGCAAG	
XC2985QEF	GATCGCATCACCATCACCATCACGGATCCGACCAGCAGCAGCTGCAAAAC	XC2985 gene for expression
XC2985QER	CAGGAGTCCAAGCTCAGCTAATTAAGCTTAGTGGTCGACGTTCAGTCGAC	
XC4208QEF	GATCGCATCACCATCACCATCACGGATCCGCGCTGATCTGCCTCTGCAC	XC4208 gene for expression
XC4208QER	CAGGAGTCCAAGCTCAGCTAATTAAGCTTAGTAACTGAGCGAGCGACCAC	
XC2481QEF	GATCGCATCACCATCACCATCACGGATCCTCCATGCATTCCCCCGTTGC	XC2481 gene for expression
XC2481QER	CAGGAGTCCAAGCTCAGCTAATTAAGCTTACAGTAGTTTTGATAGCTGCT	
XC1218QEF	GATCGCATCACCATCACCATCACGGATCCTCCCTGTCCCGCCACCGTCG	XC1218 gene for expression
XC1218QER	CAGGAGTCCAAGCTCAGCTAATTAAGCTTAGTGCAGCGAACTGCAACGTC	
XC1214QEF	GATCGCATCACCATCACCATCACGGATCCATGTTGCGTACCACGCTTGC	XC1214 gene for expression
XC1214QER	CAGGAGTCCAAGCTCAGCTAATTAAGCTTACTTACTCCTTCGGCGTGATC	
XC1708QEF	GATCGCATCACCATCACCATCACGGATCCCGCCTGTTTCTTTCCCGTGG	XC1708 gene for expression
XC1708QER	CAGGAGTCCAAGCTCAGCTAATTAAGCTTACTACGCGTTCAGTAGCTGCC	
XC4194QEF	GATCGCATCACCATCACCATCACGGATCCAGTGCACGGACACGGGTGGCA	XC4194 gene for expression
XC4194QER	CAGGAGTCCAAGCTCAGCTAATTAAGCTTAGAGTCGACGGTCTGTGACATC	
For mutation		
Xc1214E186AR	GTTCTCTACCTGCACGGCGA	Mutation of E^{186} to A^{186} of XC1214
Xc1214E186AF	*GCA*TACGGCTCCTACGACGAC	
For clone into pHM1		
QephMF	ACAGCTATGACCATGATTACGCCAAGCTTCGAGGCCCTTTCGTCTTCAC	Amplification of the gene from the respective expression plasmid pQE80l
QephMR	GACGTTGTAAAACGACGGCCAGTGAATTCCAACGGTGGTATATCCAGTG	

*All primers were designed based on *Xcc* strain 8004; Restriction enzyme sites are underlined.

References

1. Daniels MJ, Barber CE, Turner PC, Sawczyc MK, Byrde RJ, Fielding AH: **Cloning of genes involved in pathogenicity of Xanthomonas campestris pv. campestris using the broad host range cosmid pLAFR1.** *EMBO J* 1984, **3:**3323-3328.
2. Simon R, Priefer U, Puhler A: **A Broad Host Range Mobilization System for In Vivo Genetic Engineering: Transposon Mutagenesis in Gram Negative Bacteria.** *Nat Biotech* 1983, **1:**784-791.
3. Schafer A, Tauch A, Jager W, Kalinowski J, Thierbach G, Puhler A: **Small mobilizable multi-purpose cloning vectors derived from the Escherichia coli plasmids pK18 and pK19: selection of defined deletions in the chromosome of Corynebacterium glutamicum.** *Gene* 1994, **145:**69-73.
4. Huynh TV, Dahlbeck D, Staskawicz BJ: **Bacterial blight of soybean: regulation of a pathogen gene determining host cultivar specificity.** *Science* 1989, **245:**1374-1377.
