# Supplementary material for: Genome-Wide Analysis of β-Galactosidases in Xanthomonas campestris pv. campestris 8004
Source: Front Microbiol. 2018 May 11;9:957. doi: 10.3389/fmicb.2018.00957 (PMC5958218; doi:10.3389/fmicb.2018.00957)
Supplement: Supplementary file 2 [file Image_1.pdf]

GH2

```
                        *        20         *        40         *        60         *        80         *       100         *       120
Ecoli_lacZ : MTMITDSLAVVLQRRDWENPGVTQLNRLAAHPPFASWRNSEEARTDR--PSQQLRSLNGEWRFAWFPAPEAVPESWLECDLPEADTVVVPSNWQMHGYDAPIYTNVTYPITVNPPFVPTENP :
XC_2985    : --------------MDQQQLQNTLGHRLVVGERQLLRFPAQQRSAAGDRLHAPSVYRR--------ACIASLRSRQACYR-HARRRCRQQEGAAMTGINRRELLRGMLASGVRAALPAG-AA :
XC_4208    : -----------MTPAVMQQQATTAAQRVSERGVGVAMTGAMRLRVVLYRLCRRARDGR--------PGTTQQPLQRSLRAGCMALICLCTTAGVVTAAEPTNAPRERISLNADWRFRRGDPP :
XC_1218    : -------------MSLSRHRRVPAPARLGLALAFAMSQAWAAPPTAVTLDSGWQVRLVP-----------GQEQAKTYPKAAAWLPAQVPGVVQTDLIAAKVVPDP-FYRDNEGKIQWAGLS :
XC_1003    : ----------------MGGWQLAAAPDLGTRGGAELSRGAAP-------------------------------------SSSAWHVATVPGTVLTTLVDRGVYPDPDIGLNNMAIPESLSRQ :
XC_4194    : -------------------------------------------------------------------------------------------------MSARTRVALIAALGVLLAPSHAAAA :

                      *       140         *       160         *       180         *       200         *       220         *       240
Ecoli_lacZ : TGCYSLTFNVDESWLQEGQTRIIFDGVNSAFHLWCNGRWVGYGQDSRLPSEFDLSAFLRAGENRLAVMVLRWSDGSYLEDQDMWRMSGIFRDVSLLHKPTTQISDFHVATRFNDDFSRAVLE :
XC_2985    : GALLPGSADAAPAAAKDATPLAPLGDAS-----LAPRERLLFDFGWRFHPGHASDPSRDFEFGTFQRTFAKAG-KDTATAAQLAFDDSNWQQVDLP-------HDWAVTLPFRPEPISASMT :
XC_4208    : GNTVRLEYDVRPEVVRSED--GKVADAR-----PEQAQRIDADARNVLKPWILPTANSLIADPTQRHVRPPGHPGSAVAYVQPMFDDSAWQRVDLP-------HDWAIAGPFLADGPHGGMG :
XC_1218    : DWQYQTRFTVDAATLAREHVELVFDGLDTFAEVTLNGKPLLSADNMFRQWRVDAKALLKRGDNVLEVKLFSPIKKIQP-------------------------------------------- :
XC_1003    : DWWYRSTFELPASTQGK-RLELVFNGINYAGEIWVNGVQVGRTRGAFARGRFDVSSYLTPGRNAIAVRVSPPPHPGIAHEQSMTAGVGENGGMQALDG------------------------ :
XC_4194    : DAPLLHALFQPHMVLQRDAPIRLWGDAP-----------------------AGEQVTLRLGEQQVQVRADRQG------------------------------------------------- :

                    *       260         *       280         *       300         *       320         *       340         *       360
Ecoli_lacZ : AEVQMCG---ELRDYLRVTVSLWQGETQVASGTAPFGGEIIDERGGYADRVTLRLNVENPKLWSAEIPNLYRAVVELHTADGTLIEAEACDVGFREVRIENGLLLLNGKPLLIRGVNRHEHH :
XC_2985    : EEDPAAAHGYKALGTSFPENSVGWYRRMLQIPASDLGKRICLVFDGVFRDCVVFCNGHIVGRNASGYCGFEVDLSEVLDYGKPNLIVVRVDATLGEGWFYEGAGIYRHLWLQKTDPLHVPQH :
XC_4208    : ---------------RLPSWGVGWYRKALQIPASDRGRSLFLDLDGAMSYATVWLNGKLVGGWPYGYTSWRVDLTPYVVPGGHNQLTIRLDNPPDSARWYPGGGLYRNVWLTKTGPLRVAQW :
XC_1218    : -----------------------WLAKQPYALPGAYDS--------------------------------AFGDEPESRHSSTYVRKAPYNFGWDWGPRMVNAGIWKDVRVEAWDAVRVDGL :
XC_1003    : -------------PTFIASEGWDWIPAVRDRNAGLWQDVQLHATGPLAVGDTHVVTAKLAP----GHRRAELEIAVPLRNTSGTAVQGTVQLAFGDVRIQREVNVPAGGTTLTLTAADTPEL :
XC_4194    : ----------------------HWQARLPARAAG--------------------------------------GPYTLSARSAGGVTQQLDDVLVGDVWLCSGQ-SNMELQVHRTLDSRSEIA :
                                    w

                  *       380         *       400         *       420         *       440         *       460         *       480
Ecoli_lacZ : PLHGQVMDEQTMVQDILLMKQNNFNAVRCSHYPNHPLWYTL--------CDRYGLYVVDEANIETHGMVPMN---RLTDDPRWLPAMSERVTRMVQRDRNHPS-VIIWSLGNESGHGANHDA :
XC_2985    : GVFVRS-SVQGDTATAQLSTEVRNDGTASRQCVVQARITAP-------YGRIVAQAASAAVTVAPGQVQVVEQTVPLGQAALWS---IDTPQLYTLTTSVHSAGVAVDALVTPFGVRSIAFD :
XC_4208    : GTQLSTPQVSAERAQVQLAVQLDNAGAAPVQAQVSTALYALDDASGLRRGPAVAQIAAVQVQVAAGGNARVQGSTQIEAPRRWGPPPTQQPHRYVAVTEVSQGGRVVDRVETPFGVRSVVFD :
XC_1218    : HIAQQRVDADSAQLQAQLELQAGRSGPVQVNLDVLG-------------PNGQRVGQFTQDAVVDPGQNRIDLAVRIAKPKRWFPAGYGAQDRYTFVATVRDADGDSQQIKRITGLRSVELR :
XC_1003    : VVTNPRLWWPNGYGEPSLYTLQVGVAAGGVQSDLQQLRFGIR-------EVTYELSLFDERGALRRVLVDLNQARQRGERIVDVRHAAIRPVPGGNAQSLYPGALASPAVT---QLDDSTLA :
XC_4194    : DADHPSIRMFKVPAQSSPTPQRSFGGAAVWQP-------------------TTPETVKDFSAACYYFARELQKTVAVPMGLINASWGGSQLQAWIGDAALRAAGDDGPALDVLARYATSPTD :
                               l                                                    6                                      6

                *       500         *       520         *       540         *       560         *       580         *       600         *
Ecoli_lacZ : LYR-------WIKSVDPSRPVQYEGGG-ADTTATDIICPMYARVDEDQPFPAVPKWSIKKWLSLPGETRPLILCEYAHAMGNSLGGFAKYWQAFRQYPRLQGGFVWDWVDQSLIKYDENGNP :
XC_2985    : AQR-------GFLLNGAPLKLHGTNNH-QDHAGVGTAIPDALHAWRLRQLKSMG--------------CNAYRSSHNPATPELLALCDRLGMFVIEETRR------------MSTDPEAMAE :
XC_4208    : PDK-------GLVINGEHVPIRGVNNH-HDLGALGAAFNVRAAERQLQLLQHMG--------------ANAIRMSHNPPAPELLELTDRMGLLVVDEVFDSWEMKKTPLDFHLIFPQWHEPD :
XC_1218    : REKDKFGKSMEIVINGIPIFAKGANLIPLDAFPSRVSQARMRSTLQDARDANMN--------------MLRMWGGGHYQDDHFYEVADELGIMIWQDFMFGGAVP----PYDVEFRENTRQE :
XC_1003    : PHL-------VLRINGVRIAIKGGNWG-MDDWRKRVSRERLEPYFRLQRDAHFN---------------VVRNWVGQNTGASFFDLADEYGMLVLNDFWQSTQN----YNMEPADAALFLAN :
XC_4194    : AAP----------RWGKLWETWWRAHGDGAPWQPDAPGAWQPAPATLGAWDEWG----------------------------VPQLVGFNGMVWYR-------------------------S :
                            g             d                                                            g
```

```
                    620         *       640         *       660         *       680         *       700         *       720         *
Ecoli_lacZ : WSAYGGDFGDTPNDRQFCMNGLVFADRTPHPALTEAKHQQQFFQFRLSGQT---IEVTSEYLFRHSDNELLHWMVALDGKPLASGEVPLDVAPQGKQLIELPELPQPESAGQLWLTVRVVQP :
XC_2985    : LETMVRRGRNHPSVILWSLGNEEPQQVT-ERGARIVTRMQQRVRQLDPTRP------TTFAMDKGFGDG-VGQVVDVVG--FNYR------------------TSQMDGFHAQYPNIPIYGS :
XC_4208    : LRAMLRRDRNHPSIILWSVGNEVGEQYTGEAGAAIARRLHAIVHAEDPTRP------ATAAMNYASPEMPLPAALDVIS--LNYQGEGIRDTPEFEGTERIRKPPQYPAFHAKFPRKAILSS :
XC_1218    : AIEQVKRLRDHPSVVLWCGNNEVQTGWENWGDRVKFKQSVDPEERSRIERG----------MTTLFGTVFREVVATYDSDVPYWSTSPGTNFDGAADQANDGDMHYWKVWGGPALPVTEYLN :
XC_1003    : AAEVITRFRNHPSIVLWFGCNEGVPAPILNEGLDALVAALDGTRWYTASSN--------EVNLQGSGPYNYREPVAYFN---KLAQGFSVEVGTPSFSTLESFKASVPAVQDQWPISDSWAY :
XC_4194    : TVELTAAQAAQDATLLLGPVDELDQTWVNGHAVGSSYGADQPRRYSVPRGQ--------------LQAGRNSIVLNVLN---------------------------------TYRRGGLLGD :
                        p   l     e

                  740         *       760         *       780         *       800         *       820         *       840         *
Ecoli_lacZ : NATAWSEAGHISAWQQWRLAENLSVTLPAASHAIPHLTTSEMDFCIELG--NKRWQFNRQSGFLSQMWIGDKKQLLTPLRDQFTRAPLDNDIGVSEATRIDPN-AWVERWKAAGHYQAEAAL :
XC_2985    : ETGSTVSVRGNYQR----------------DDTRGYTRAYDTDHPWWASTAEAWWSYVAQRPYIAGGFIWTGFDYRGEPTPYNRWPNVGSQFGVLDSCGFPKDNYWYYRAQWTSEP-VLHLF :
XC_4208    : ETASALSSRGVYLFPVSAQSSAPVRDGRGGDSVAHQVSAYELHAVDFGSSADKVFAALDRHPFVAGEFVWTGFDYLGEPTPY--YSSRSSYSGILDLAGFPKDRYWLYQARWRPELPMAHLL :
XC_1218    : VTPRFMSEYGLQSFPDMRTVRAFAEPGDMDPESPVMRVHQKFDKGNGNKRLMLYIRREFGEPKDFESFVYLSQLMQAEGINLAASHLRASRPQSMGSLYWQLNDVWPGASWSSVDYYGRWKA :
XC_1003    : HDWHQSGNGDTASFMRSLSDKLGAPTSLDDFERKAQLLNYETYRAIFEGFNAQLWTKNSGRLLWMSHPAWPSNMWQIYSHDYDTHAAYYGVRNAAEPLHVQMN-------------LPGHEV :
XC_4194    : ASSRALQFADGSRVPLDRPWQYQRVPAAVGMPPRAPWSSAAGLTTLYNGMIAPLGQLGLRGVLWYQGESNAGDAAHYPALLDAWRRDWRQRFG------------------------AQLPL :

                860         *       880         *       900         *       920         *       940         *       960         *
Ecoli_lacZ : LQCTADTLADAVLITTAHAWQHQG---KTLFISRKTYRIDGSGQMAITVDVEVASDTPHPAR-IGLNCQLAQVAERVNWLGLGPQENYPDRLTAACFDRWDLPLSDMYTPYVFPSENGLRCG :
XC_2985    : PHWNWDGLLQPDDKGRVEVWCHSNLEAVELLVNGVSQG-LQQVPAYGHVEWR-VAYAPGVIEARGYRGGKLVLTERRETVGKPAAIRLSCD---------RSALRADAEDVAVVKVEIVDAQ :
XC_4208    : PHWSWPGREGQ----VTPVHVFTSGDEAELFVNGVSQGRKRKAPLQYRLRWDAVVYQPGELRVQAYKHGRPWASDRVQTAGRATRMQLSAD---------RNQIRGDGKDLSFITVRLLDAD :
XC_1218    : LHYHARRFYAPEMIAALRNDKGQT---------EVSLVSDRTTPLQARWRMRVMSMEGKVLSKREEKATLKPLSSLQVGNFSDKQLLGNADPKRS-----YAVFELLDGDTVLSREVVFFAA :
XC_1003    : VVVNNTTTAVRGLRVRAETYAADG--------RLLQQREQAVDAAAVAVSAPVLQLTPLLANAKDLGFVRLQLLERDGTVRSRNFYWVARD---------AAAMRCLDALAPVPLELTTQLQ :
XC_4194    : LVVQLANYGMPPTEPVESGWAQLR--------EAQRRFVAGDAHAGLAVAIDIGDRYDIHPANKQELGRRLAHAARHVVYGEAIAASGPVP----------RDVQRDGEALRVRFDAVDTAL :
                                                                                        r

              980         *      1000         *      1020         *      1040         *      1060         *      1080         *
Ecoli_lacZ : TRELNYGPHQWR-------GDFQFNISRYSQQQLMETSHRHLLHAEEGTWLNIDGFHMGIGGDDSWSPSVSAEFQLSAGRYHYQLVWCQK------------------------------ : 10
XC_2985    : GRLVPNADHLVHLVLRGPARLIGVGNGDPSSHEDDKAPQRKAFNGLCMALLRSTR-SSGQILLQATAPGLTSAALHLQAATSALRPSVD------------------------------- :  9
XC_4208    : GRPAPTAGDRLRFRVEGAGELVATDNGDPTNLEAFVLPERNAFNGLCLVIVRAKPGAQGPITVHVQSDTLQSASVQVLAE---------------------------------------- :  9
XC_1218    : AKQLALPTATIDSQWRADGDGYALTLSSKTLAREVWLSFGDVDAALSDNAFDLLPGEPLTVHVTSKTALAQLQSALQVRDLASTLAGAPPEPAESK------------------------ :  8
XC_1003    : AGDAPVLSATVRNPSQHVALGAKLTLVDAQGQRILPAYYSDNYLNLAPGESRTVEIRGPAEASLRAARVQLRGWNLAPSSGTASGSP--------------------------------- :  8
XC_4194    : QSYSASAPIGFEVCGAASGSCHYAPASVHGDTVTLRVP-----ADLQATRVRYCWADSPVCTLYDRSGLPAGPFELPITAATSR------------------------------------ :  6
                                                          l
```

GH35

```
                   *        20         *        40         *        60         *        80         *       100         *       120
XC_2481 : MSSLAVALAACQSGEPDSAAKPQAAATPGTTAPATAPAAAPATQTPMPQFVSKDGKHALMVDGAPFLILGAQVNNSSNYPGVMDKVWPAMQVLGPNTVQVPIAWEQVEPEEGKFDFS---FVDTLLAQ
XC_1708 : -MRLFLSRGAVR----------RCVAQVLLCMLALLGGPAVANAEELPRFFTQNGRHALLVDGAPYTILAAQLHNSSAWPAVLPPALDQVVALHANTVEAPVYWEQFEPAPGRFDTT---NVDALIAG
XC_1214 : --MLRTTL--AP--------------LVLALSIALPITATAASDDQWPTFATQG--TQFVRDGKPYQVLSGAIHFQRIPRTYWKDRLQKARALGLNTVETYVFWNLVEPQQGQFDFNANNDVAAFVRE
Xam_Bga : --MLRTTL--AP--------------LVLALALALP--AAAATPESWPTFGTQG--TQFVRDGKPYQLLSGAIHFQRIPRAYWKDRLQKARALGLNTVETYVFWNLVEPQQGQFDFSGNNDVAAFVKE
             L   l                   vl    Al   aa A     P F 3q      6 DG P5 6L   6h            l    aLg NTV2  6 W  vEP  G FDf     V a 6

           *       140         *       160         *       180         *       200         *       220         *       240         *
XC_2481 : AREHKVRLVL----LWFATWKNNGPAYAPHWVKTDN-----QRFPRVVTREGKAIGSLSPHAQATLDADRKAFVAFMQHLKQVDP-QHTVIMIQ--PENEPGTYGSVRDFSPMAQKLFDGPVPDELLK
XC_1708 : ARKRGLRVAL----LWFGSWKNGQMHYVPEWIKRDE-----ATYPRMRDANGEPVDVLSPHVAANVQADARAFTALMQHLRKVDGDRHTVIVVQ--VENEPGAIGTVRDHGPAGEAAFAQPVPAGIAA
XC_1214 : AAAQGLNVILRPGPYACAEWEAGG---YPAWLFGKDNIRIRSRDPRFLAASQSYLDAVAQQVRPLLNHNGGPIIAVQVENEYGSYDDDHAYIADNRAMFVKAGFDKALLFTSDGADMLANGTLPGTLA
Xam_Bga : AAAQGLNVILRPGPYACAEWEAGG---YPAWLFGKGNIRVRSRDPRFLAASQAYLDALAKQVQPLLNHNGGPIIAVQVENEYGSYADDHAYMADNRAMYVKAGFDKALLFTSDGADMLANGTLPDTLA
          A   g6 6 L       a W  gg    P W6          r PR   a    6d 6   v   6  1     A                6                 f   g    a       la

           260         *       280         *       300         *       320         *       340         *       360         *       380
XC_2481 : RLNKQPGSWAQVFG-----ADADEIFHAWSIGRYIDQVAEAGKQAYPLPMYVNAALRGPFNPGQPGQYASGGPTDNVLDVWKAAGPHIDLLAPDIYMPEYRMYTTVLERYARPDNALFVAETGNRPEY
XC_1708 : ALGKPAGSWQQLFG-----AEAAEAFNAHATAAYIEQVAAAGKRVYPLPLYVNTWLRYKGKRYPGMDYPSGGATVNVFALWRAATPSIDFIGTDIYTSDYGEYTKVIGQYARPDNPAWVSETGFEAAT
XC_1214 : VVNFAPGEAKSAFDKLIKFQPDQPRMVGEYWAGWFDHWGTPHASTNAKQQTEELEWILRQGHSANLYMFIGGTSFGFMNGANFQGNPSDHYAPQTTSYDYDAILDEAGRPTPKFALMRDVITRVTGVQ
Xam_Bga : VVNFAPGEAKSAFDKLIKFRPDQPRMVGEYWAGWFDHWGKPHAATDARQQAEEFEWILRQGHSANLYMFIGGTSFGFMNGANFQNNPSDHYAPQTTSYDYDAILDEAGHPTPKFALMRDAIARVTGVQ
           6n  pG     F                  a 5 d                                  GG 3              D  ap     dY       g             t

               *       400         *       420         *       440         *       460         *       480         *       500         *
XC_2481 : ARYLFPTLGHDGVGWSAFGMDYTRYSNYPLGAKHVNEETLAPFALGFKLVSL--------------GMRPFAKAASEGKLHGTAE---EPGQAVQELKLNDRWSATITYGVPQFWFK-GEPPGNPEP
XC_1708 : APYLFHVLGQGGIGFSVFGIDGNPDS-------EANRAAIAAHAANFRQLAP--------------LQRLIAQANLDGRLQAVAE---QPGSPQRTLRFGD-WEAKVSFGAPLWGDAPAILPGNDDH
XC_1214 : PPALPAPIAMAALKDAPLRESASLWDNLPAPIAIDTPQPMEHFGQDYGYILYRTTVTGPRKESLYLGEVRDVARVYVDQKPVGSVERRLQQVATEVDIPAGQHTLDVLVENSGRINYGPRMADGRAGL
Xam_Bga : PPALPAPITTTTLPATPLRESASLWDNLPTPIAIDTPQPMEQFGQDYGYILYRTTITGPRKGPLYLGDVRDVARVYVDQRPVGSVERRLQQVSLEVEIPAGQHTLDVLVENSGRINYGTRMADGRAGL
           p L   6    6             n p          6  f   5  6                   R  A    d 4  g  E   2       6  g      6               G

               520         *       540         *       560         *       580         *       600         *       620         *
XC_2481 : IGAALIAELGPDEFLVTGYHVR-----VTLHPASEATANMLYDRVEEGQYDGDTWQFQRNWNGDQTDYGVNFS------SAPQLLKIKLATYN------------------------------- : 5
XC_1708 : AGRLLVAQLGPEEFLVTGIAAR-----IEFFRSAADTRHGQLLQVEQGRYVDGRWQMERQLNGDQTDYGLNFGRTDAAGQPPPVLRVRVGSY-------------------------------- : 5
XC_1214 : VDPVLLDNQQLTNWQAFPLPMRSPDSIRGWTRNTVEGPAFHRGNLRIGTPADTYLDMRAFGKGIAWANGVNLGRHWNIGPQRALYFPAPFQRKGDNTVVVFDLDSTAKPSVRGLQQQVWITPKE : 6
Xam_Bga : VDPVLLDSQQLTGWQAFPLPMRTPDSIRGWTGKAVQGPAFHRGTLRIGTPTDTYLDMRAFGKGFAWANGVNLGRHWNIGPQTALYLRPSSAR--VTTRWWSSTWTMLHPSVRG----------- : 5
              L6       5       R                      6  G   d    m     G     G6N gr    g    6
```

Figure S1. The alignment of *Xcc* GH2 and GH35 family proteins

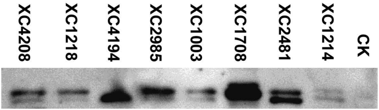

Figure S2. GH2 and GH3 proteins expressed in *E. coli* detected by western blotting

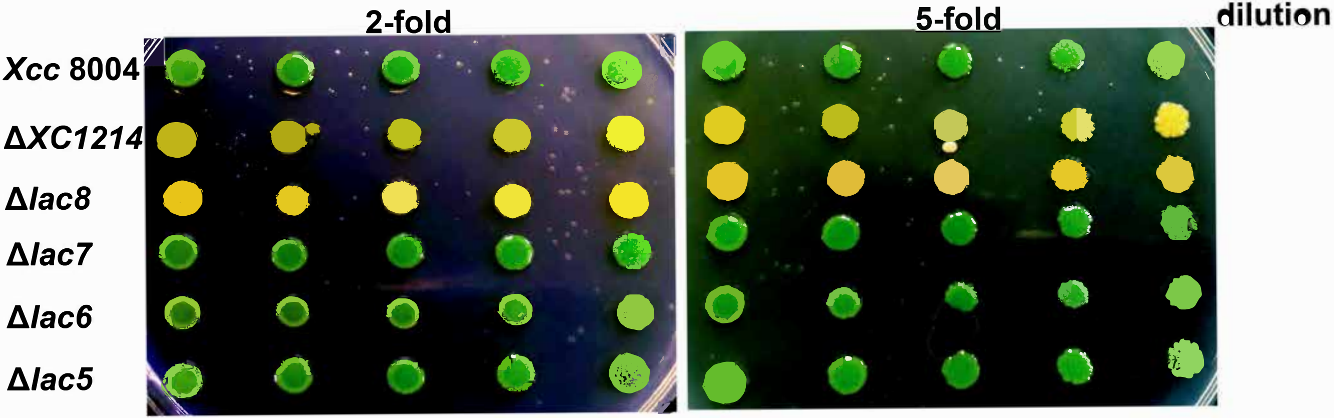

Figure S3. The enzyme activities of GH2 and GH35 mutants of *Xcc* 8004 on X-gal containing plates

Each spot was inoculated with 2 μl of a 2- (left) or 5-fold (right) dilution series from cells ($1*10^5$ cells/ml)
